# Intimate partner rape: do rape myths still influence verdict outcomes when the defendant is an ex-partner?

**DOI:** 10.3389/fpsyg.2026.1778367

**Published:** 2026-04-15

**Authors:** Caroline Lilley, Dara Mojtahedi, Dominic Willmott

**Affiliations:** 1 School of Law, Faculty of Arts and Humanities, University of Sheffield, Sheffield, United Kingdom; 2 Department of Psychology, Faculty of Health and Social Sciences, University of Huddersfield, Huddersfield, United Kingdom; 3 Department of Criminology, Sociology and Social Policy, School of Social Science and Humanities, Loughborough University, Loughborough, United Kingdom; 4 Faculty of Psychology, SWPS University, Wroclaw, Poland

**Keywords:** intimate partner rape, juror attitudes, jury decision-making, rape myths, rape trial

## Abstract

**Introduction:**

Despite research highlighting the influence of rape attitudes and other juror traits on trial outcomes, few studies have examined such relationships within intimate partner rape trials, prioritising instead decision-making in so-called “date rape” cases. The current study, therefore, sought to investigate the relationship between juror demographic traits, their pre-trial legal attitudes, and rape myth beliefs, upon subsequent verdict decisions made in an intimate partner rape trial.

**Methods:**

The study adopted a mock trial paradigm, with methodological enhancements aimed at increasing ecological validity. Mock jurors (*N* = 435) completed a series of attitudinal and demographic questions online before observing a recreation of a genuine intimate partner rape trial and subsequently rendering their verdict.

**Results:**

Results revealed that ethnicity, educational attainment, and rape myth acceptance, though not varied legal attitudes, were all significant predictors of the verdict selections that jurors made. Caucasian, university-educated mock jurors and jurors who rejected rape myths to a greater extent were those most likely to find the defendant guilty. Female jurors were also significantly more likely to return a guilty verdict before, though not after, controlling for variation in rape myth beliefs.

**Discussion:**

These findings offer further support to the wealth of existing literature that suggests jurors' pre-trial rape myth beliefs, alongside other demographic characteristics, appear to predispose juror judgements and decision-making, and extend upon past literature in identifying a similar trend within intimate partner rape trials. Findings highlight the need for targeted juror reforms, such as myth-debunking juror education, before such recommendations are made. Before such recommendations are made, further enhancements to mock-trial procedures to maximise ecological validity, alongside greater research among genuine trial jurors, are warranted.

## Introduction

### The prevalence of sexual abuse

Global statistics unequivocally evidence a significant and pervasive epidemic of sexual violence against women (World Health Organisation, 2024). Current estimates suggest that 736 million women worldwide will experience some form of sexual violence at least once during their lifetime, representing nearly one-third of the global female population (UN Women, 2025). Within England and Wales (E&W), national data reveals a concerning prevalence of sexual violence: as statistics report, approximately one million individuals experienced sexual violence in the year ending March 2024 (Office for National Statistics, 2024). Notably, 36% of those sexual offenses were classified as rape (Office for National Statistics, 2024). Whilst acknowledging that both men and women can be victims of sexual violence, crime reports continue to highlight the significant gender disparity in its occurrence. Women and girls are disproportionately affected, with data from the Office of National Statistics (Office for National Statistics, 2021) indicating that women are around four times more likely to experience sexual violence than men. However, it should be acknowledged that the underreporting of sexual assaults significantly impacts the accuracy of published prevalence rates, potentially leading to a considerable underestimation of the true extent of the issue.

A substantial majority of this violence is perpetrated by current or former intimate partners, with over 640 million women aged 15 and older having experienced some physical or sexual violence at the hands of an intimate partner (UN Women, 2025). Global prevalence data further emphasizes the disproportionate burden of intimate partner rape (IPR) on women. The World Health Organisation (2024) reports that 27% of women aged 15–49 who have been in an intimate relationship have experienced sexual violence at the hands of their partner. Available recent statistics for England and Wales indicate that 44% of rape victims were assaulted by intimate partners (current or former), 37% who were raped by someone other than an intimate partner to whom they were already acquainted, with the remaining minority of recorded rapes perpetrated by strangers (Office for National Statistics, 2021). Importantly, in this paper we differentiate intimate partner rape (IPR) from traditional labels of “date rape” or “marital rape” in seeking to highlight that these narrow conceptions often mean that rapes that occur by intimate partners outside of stable, formalized and legally recognized relationships are often overlooked. Therefore, as many rapes occur outside of traditional date rape scenarios and within intimate relationships where a formal partnership or marital relationship does not exist, the focus on this paper will be investigating juror decision making within an IPR case.

### Intimate partner rape

Intimate partner rape (IPR), in its widest conception, can be defined as rape that occurs between current, former, cohabiting, or dating partners, irrespective of any formal and agreed label which recognized the relationship as such. Unlike stranger rape, which is stereotypically linked to physical violence by the perpetrator and resistance by the victim, most likely in an outdoor setting (Temkin and Krahé, 2008), IPR is characterized by its occurrence within established or less formalized relationships where a history of intimacy (sexual or non-sexual) often already exists (Jaffe et al., 2021). Because IPR frequently happens in private residences without witnesses or evidence, such as CCTV, it often bypasses typical societal interpretations of rape.

IPR is rarely an isolated incident; it is more accurately viewed as part of broader patterns of intimate partner violence (IPV) used to maintain power and control (Bagwell-Gray et al., 2015). Perpetrators may use sexual violence as a means to uphold dominance, humiliate intimate partners, or exert control interpersonal interactions (Lilley et al., 2023; Kirkman et al., 2025). The psychological impact of IPR is uniquely profound. Contrary to a popular belief expressed by some, that being raped by someone you know is less traumatic than if the offender was a stranger, research among IPR victims indicates comparable and/or heightened rates of emotional distress and psychological trauma following the sexual abuse (Ansara and Hindin, 2011; Campbell, 2002; Shields and Hanneke, 1992; Temple et al., 2007; Torrioni et al., 2025).

### Rape attrition in the criminal justice system

Despite legal reforms that previously amended outdated definitions and legislation, issues with attrition of rape cases throughout the CJS have only improved slightly. Under-reporting of offenses remains a significant contributor to poor attrition rates. That being said, the popularity of the #MeToo movement and the large-scale media coverage of prosecutions against high-profile individuals has led to an increase in willingness to report sexual offenses (see Willmott et al., 2021). Yet, rape and sexual assault remain the least likely to progress from perpetration to conviction compared to any other criminal offense (Hester and Lilley, 2017). Notably, those committed by an ex/current intimate partner display significantly higher rates of attrition than other forms of sexual violence (i.e. stranger rapes) (Hohl and Stanko, 2015; Lea et al., 2003). In recent years, despite record highs of reported rapes, few resulted in charges. In 2021–2022, of 70,330 recorded rapes, only 2,223 resulted in charges (Rape Crisis England Wales, 2024). In a previous year (2019–2020).

Literature continues to show that four core stages reliably account for rape case attrition: *(1) victim withdrawal of allegations, (2) police unwillingness to proceed, (3) prosecutor charging decisions and (4) juries returning not-guilty verdicts at trial* (for a detailed discussion see Willmott et al., 2021). While the exact stage at which attrition occurs the most remains the subject of ongoing debate, there is a consensus that extra-legal factors, such as rape myths and rape stereotypes, significantly contribute to attrition throughout the entire criminal justice process (Brown et al., 2007; Spohn and Tellis, 2019; Willmott and Hudspith, 2024). Research maintains that criminal justice practitioners, from police officers to judges, are susceptible to the influence of rape stereotypes, myths and normative sexual scripts (Hester and Lilley, 2017; Nørgaard Madsen and Mikkelsen, 2024; Willmott and Woodhams, 2026). Endorsement of which ultimately affects legal decision making; from which cases progress through the criminal legal system to whether defendants are deemed culpable and complainants believed (Curley et al., 2026; Headd and Willmott, 2026; Weare et al., 2025). Consequently, cases that deviate from stereotypical “real rape” scenarios, such as those involving intimate partners, are more likely to be dismissed or otherwise “fall out” of the CJS (O'Neal et al., 2015; Spohn and Tellis, 2019), further exacerbating attrition rates in non-stranger rape cases.

### Rape myths, legal attitudes, and juror decision making

The criminal legal system relies on jurors as “judges of fact” (Ingman, 2011). The expectation is that jurors will approach their role free from preconceived notions or bases, often idealized as “blank slates”, to ensure fair and impartial verdicts (Louden and Skeem, 2007). However, this idealized notion undoubtedly falls short of reality. Opposing conclusions from identical case information (e.g. as reported in Willmott et al., 2018) suggest jurors base their decisions on more than trial evidence alone. A likely explanation, as supported by the literature, is that pre-existing attitudes, beliefs and characteristics influence juror decision-making in criminal trials. Attitudinal bias can encompass broad, general attitudes regarding a group of individuals, specific circumstances or a certain environment, or it can have a particular focus (for example, rape myths bias people's opinions about the victim, perpetrators and circumstances of rape). This paper will primarily focus on rape myths as an example of crime-specific bias. Still, it will also explore general criminal justice biases and their effects on juror decision-making.

The Story Model of juror decision-making (Pennington and Hastie, 1992), supported by empirical research (Pennington and Hastie, 1988; Willmott et al., 2018), posits that jurors are not merely passive recipients of facts; rather, they are active participants who construct a cohesive narrative from trial information. These stories are built by blending trial evidence with jurors' own pre-existing knowledge and extra-legal biases. Critically, when key information is missing, jurors bridge these gaps with personal inferences and existing societal beliefs (Pennington and Hastie, 1988). Trials that lack compelling evidence or objective markers, such as DNA evidence or third-party witnesses, are particularly vulnerable to these biases. This is a defining characteristic of rape cases in England and Wales, especially that of IPR, which typically center on consent disputes behind closed doors. In the absence of clear-cut evidence in these cases, jurors may turn to pre-existing notions of rape to determine the guilt of the defendant, making the subjective beliefs of the jury as influential as the evidence itself. In the context of sexual violence, such attitudes are dubbed rape myths.

Rape myths have been identified as factually incorrect, prejudiced beliefs that determine how information from a rape case is processed and interpreted (Bohner et al., 2005; Dinos et al., 2015). Rape myths are defined as false beliefs that attempt to transfer the blame onto the victim by (1) blaming the victim, (2) excusing the offender, (3) doubting the allegations, or (4) believing rape is exclusive to a specific type of individual (Suarez and Gadalla, 2010; Lilley et al., 2023; Bohner et al., 2009). A recent survey of more than 3000 UK adults, the largest on this topic in the last 5 years, found that whilst the public's accurate understanding of rape has grown over recent decades, there are still significant false beliefs, misunderstandings and a lack of knowledge surrounding rape (Crown Prosecution Service, 2024). The presence of such inherent beliefs in society raises serious questions about the ability of jurors to handle complex and often ambiguous evidence in sexual assault cases. Numerous studies have evidenced a relationship between high rape myth acceptance and not-guilty verdicts (McGee et al., 2011; Hammond et al., 2011; Maeder et al., 2015; Willmott et al., 2018).

Raising doubts about the credibility of rape victims and their version of events is a common function of rape myths and one that is frequently applied and observed within the courtroom. Empirical work has reported that lawyers, judges and even jurors routinely draw upon these myths to make sense of the evidence presented at trial (Eyssel and Bohner, 2011; Hammond et al., 2011; Klement et al., 2019; McKimmie et al., 2014; Parsons and Mojtahedi, 2022; Smith and Skinner, 2017; Süssenbach et al., 2013; Weare et al., 2025). Given the abundance of research that evidences the existence of rape myths across societies, communities, and cultures, it is reasonable to assume that random selection of jurors from these communities is likely to result in rape myths being present at trial. Researchers have identified the same pattern across jury investigations. Moreover, studies demonstrate a relationship between rape myth acceptance and not-guilty verdicts (Hammond et al., 2011; Krahé et al., 2007; Leverick, 2020; McKimmie et al., 2014; Sommer et al., 2016; Willmott et al., 2026a). A likely explanation for this relationship is the interpretation of case information through rigid gender stereotypes and “real rape” schemas (Millar et al., 2025). “Real rape” stereotypes perpetuate the misconception that rape exclusively involves strangers in a specific context, typically involving violence or the use of a weapon. This distorted perception leads jurors to devalue cases that deviate from this narrow definition, such as those involving intimate partners (Ellison and Munro, 2009, 2013). As a result, cases that do not conform to real rape stereotypes are less likely to progress to conviction, contributing to higher attrition rates observed in non-stranger rape cases. Mock juror simulations have shown that jurors rely heavily on what they perceive to be normal sexual conduct and misguided beliefs about “real rape” when determining guilt (Ellison and Munro, 2009, 2013). Therefore, it can be assumed that low conviction rates may stem, in part, from jurors' inability to impartially evaluate complex evidence. Furthermore, the oversimplification of non-stranger rapes, often attributed to stereotypes and perceived circumstances of “real rape”, contributes to this issue (Sommer et al., 2016). A likely explanation is that many people's expectations of rape do not accurately reflect reality (see Willmott et al., 2026a for a detailed discussion).

IPR is a prime example of an offense that contradicts the “real rape” stereotype and pervasive public perceptions of sexual violence. Because it often occurs within the context of established or historic sexual relationships, IPR is often obscured by general, broad rape myths and specific situational myths. As noted in previous research, IPR myths typically center on gendered expectations regarding a woman's role and behaviors within a relationship, which are then used to rationalize sexually abusive behavior between partners (Bergen and Bukovec, 2006; Hill and Fischer, 2001; Pemberton and Wakeling, 2009). The double effect of both general and situational myths creates considerable hurdles for prosecution; since the act does not align with stereotypical narratives of rape, it is often not recognized as a genuine allegation (O'Neal et al., 2015; Osborn et al., 2018).

Most importantly, the existence of a previous sexual relationship can significantly skew perceptions of consent. Unfortunately, for cases such as IPR, mock jurors have been found to rely heavily on their interpretations of consent when deciding upon a rape case (Ellison and Munro, 2009, 2013). Juror participants across all deliberations in Ellison and Munro's rape case simulations agreed that consent would not be discussed openly between partners but instead would be expressed through non-verbal, implicit clues (Ellison and Munro, 2009, 2013). Throughout mock deliberations, the topic of consent is typically discussed in relation to the complainant rather than the defendant's interpretation of gaining consent. This is because women are frequently perceived as responsible for communicating their willingness or refusal (Ellison and Munro, 2009, 2013). Jurors in these cases may struggle to differentiate past consensual intimacy from the specific instance of non-consensual assault, leading to the belief that prior sexual history implies a “blanket consent”. Consequently, IPR is typically interpreted as miscommunications between partners; it is noted that a closer victim-perpetrator relationship increases the blame attributed to the victim (O'Neal et al., 2015; Osborn et al., 2018). A likely explanation for this may be the ambiguity surrounding reasonable belief in consent when presented with a complex rape scenario (Osborn et al., 2018).

More generally, however, literature implies that all verdict decisions, regardless of criminal offense type, will likely be impacted by how an individual perceives the criminal justice system. To accurately measure the extent to which jurors hold biases against the criminal justice/legal system, Lecci and Myers (2008) developed the Pre-Trial Juror Attitudes Questionnaire (PJAQ). The scale was designed to identify jurors who may be predisposed to certain verdicts or who may have difficulty remaining impartial during a trial. The PJAQ consists of 29 items that measure attitudes across six key subscales.

The first, conviction proneness, gauges a juror's inclination to favor conviction over acquittal. In these instances, individuals will favor the prosecution's adaptation of evidence and condemn the defendant (Springer and Lalasz, 2014). Literature identifies this as a highly persuasive factor against verdict decisions, alongside confidence in the criminal justice system (CJS) (Lecci and Myers, 2009). The system confidence subscale measures the level of trust and confidence a juror has in the criminal justice system. It assesses perceptions of fairness, impartiality and the validity of the legal process. For example, research by Farrell et al. (2013) found that greater trust toward police increased jurors' likelihood of convicting defendants. However, greater trust in the courts had an inverse effect on verdict decisions. Both conviction proneness and system confidence have been identified as significant predictors of guilty verdicts (Lecci and Myers, 2009). That being said, very little research exists that has explored such associations.

The Racial Bias subscale examines racial prejudice and stereotypes that may influence a juror's perceptions of defendants, complainants and witnesses in the same manner that the Innate Criminality subscale measures beliefs about the inherent criminality of certain individuals or groups based on their appearance or perceived characteristics. Both these subscales found that when a defendant had noticeable characteristics that are typically described as “criminal” (i.e. they were Black, had tattoos, scars or piercings), they were more likely to be viewed as guilty and receive harsher sentences (Johnson and King, 2017; Mitchell et al., 2005). Though notably, some recent research (Curley et al., 2026) found no such effect of defendant race in a mock male-on-male rape trial meaning the role of pre-trial racial bias remains somewhat unclear in jurors rape trial decision making.

This PJAQ also considers jurors' perceptions of the defense. The “Cynicism toward the defense” subscale explores jurors' negative attitudes toward the defense and their strategies for appealing criminal convictions. It explores jurors' beliefs about the underlying motivations of defense lawyers and their willingness to manipulate the system (Lecci and Myers, 2008). Research has already found that jurors who are cynical of a defendant's account are less likely to find them credible and, ultimately, are more likely to favor the prosecution (Sandys et al., 2018; Willmott et al., 2018). In particular, defense arguments that contradict the personal values of a juror are far less likely to be viewed as credible (Higgins et al., 2007). The final subscale of the PJAQ, Social Justice, measures a juror's beliefs about the fairness, equality and the role of the justice system in addressing social issues. It assesses attitudes toward phenomena like socioeconomic disparities and the rights of the accused. The PJAQ has been extensively validated (see “Methods” for further discussion) and is widely used in psycho-legal research. The scale helps to identify potential juror bias, providing invaluable insights into the factors that may influence juror decision-making. As each subscale has been previously situated in the wider literature, the PJAQ has been deemed relevant to the current study.

Overall, however, although prior research consistently demonstrates that heightened rape myth acceptance predicts not-guilty verdicts, mock jury research tends to exhibit some methodological limitations. The inability to experimentally research real-world jurors has undoubtedly impacted the investigation of criminal trial decision-making, yet researchers have drawn from numerous methodological approaches to do so. By far, the most popular method of jury research is mock jury simulations, which aspire to reflect the processes of genuine criminal trials. These simulations allow researchers to experimentally manipulate variables with more control than possible in a real-world courtroom (Willmott et al., 2026b). This method of jury research also allows for greater control over independent and extraneous variables. However, such simulations are not without limitations. One example of a limitation is the use of fictional case materials and artificial environments. This significantly reduces the ecological validity of any findings produced and limits applications to real-world legal settings. Case information presented in a written vignette allows mock jurors to approach case information from an idiosyncratic view (Dinos et al., 2015). Some research indicates that participants attribute less blame to the victim when trial information is presented as a video rather than a vignette (Sleed et al., 2002). Another limitation often subscribed to mock jury research is the use of unrepresentative student samples. Mock juror samples that do not reflect the community in which natural jurors are selected limit their application to real jurors within the CJS due to their lack of diversity exhibited in the general population (Willmott and Hudspith, 2024). The present study sought to build upon these concerns. For a detailed description of how, please see the method section below.

### Demographics and juror decision making

A considerable amount of previous research has attempted to identify the relationship between juror demographics, particularly age and gender, and verdict decisions, yielding small or inconclusive results (Devine and Mojtahedi, 2021; Higgins et al., 2007; Anwar et al., 2014). Early research concerning age concludes that older jurors are more likely to return a guilty verdict than their younger equivalents (Sealy, 1981; Ruva and Hudak, 2013). Hence, guilty individual *juror* verdicts may increase in correlation with juror age (as evidenced by Anwar et al., 2014). Explanations for this relationship cite more favorable perceptions of law enforcement and criminal justice institutions, more obedience to authority, lesser use of technology, and less exposure to misinformation (Sealy, 1981; Ruva and Hudak, 2013).

However, some research fails to obtain any significant evidence of such associations (Ruva and Hudak, 2013). Potential explanations for contradictory findings suggest differences in methodological approaches, differing sample sizes, and employed measures, all of which contribute to inconsistent results, as typical of most experimental research.

With regards to juror gender, prior research indicates that female jurors are significantly more conviction prone than male jurors when concerned with crimes of a sexual nature, as these crimes are likely to be disproportionately gendered toward female victims (McCoy and Gray, 2007; Rape Crisis England and Wales, 2019). Previous research has found that juror gender is a direct predictor of verdict outcomes, with females 6.67 times more likely to return a guilty verdict than their male counterparts (Pettalia et al., 2017). Female jurors make more pro-victim decisions than males regarding victim credibility, guilt judgements and empathy (Bottoms et al., 2011, 2014). Alternatively put, existing literature suggests male jurors are significantly less likely to return guilty verdicts in rape cases, regardless of variation in the strength of the evidence and nature of the case, when compared to their female juror counterparts. Likewise, recent research suggests that it is female jurors who are those most likely to change their verdict decision after group deliberation; from guilty to not guilty whereas male jurors rarely shift from their initial not guilty pre-deliberation verdict reference (Willmott and Woodhams, 2026). Nevertheless, existing juror-gender literature exhibits considerable variation, with many studies finding little or no differences between the biological juror sex and final verdict outcomes (Willmott et al., 2026a). Inconsistent associations between gender and verdict are particularly evident within ambiguous cases where this a lack of strong physical or witness evidence in the case (Lynch et al., 2019). Further research is required to examine the role of juror gender on final verdict decisions specifically within rape cases where gender asymmetry in victimization and perpetration remains (Kirkman et al., 2025). For a recent review examining differences in juror decision making based upon gender, see (Millar et al. 2025).

In contrast, a large body of research has established a direct relationship between ethnic background and overall verdict decisions. Generally, research indicates a same-race leniency bias that implies jurors favor defendants of similar ethnicity and are more likely to determine a different race defendant as guilty (Bottoms et al., 2004). Yet, opposing research reports finding little or no direct relationship and is, in fact, dependent upon other characteristics, such as physical attractiveness, socio-economic status and crime-specific stereotypes (Sommers, 2007; Maeder et al., 2015). Unfortunately, due to an exceedingly white-dominated legal system, literature tends to focus on white/black individuals rather than being inclusive of all minority groups, as these groups are underrepresented within the genuine criminal justice system (Sommers, 2007; Esqueda et al., 2008). Despite apparent interest in the effects of juror ethnicity, research is still primarily concerned with defendant/victim race, particularly in sexual assault cases where ethnic stereotypes suggest minorities are more likely to be perpetrators (Maeder et al., 2015).

Finally, regarding juror educational attainment, research is somewhat limited, perhaps indicative of a publication bias. Since the beginning of jury research, researchers have questioned the reliability of student samples in accurately determining what juror biases impact verdict decisions (McCabe et al., 2010). It is argued that student samples do not accurately reflect the jury pool, which consists mainly of older, less educated members of the public (DeMatteo and Anumba, 2009). Nonetheless, research by Hosch et al. (2011) identified several potential influences of higher educational attainment on juror decision-making. For instance, individuals with higher education make more formulated, informed decisions, are compliant with authority and demonstrate a higher self-regard (Hosch et al., 2011). However, this research focused on comparing student and community samples rather than the effects of educational attainment itself. Willmott (2018) in testing the direct effect of educational attainment on juror decision making in an acquaintance rape trial found no effect on verdict selections. Thus, additional contemporary research is required to understand the full effects of higher educational attainment on juror decision-making and in varied rapes of rape trials including, IPR.

### Study rationale

Existing research on the influence of juror characteristics on verdict decisions often yields inconsistent results, with significant disagreement regarding the predictive power of various juror traits, attitudes and demographics. Therefore, the present study aims to investigate the relationship between legal attitudes, modern rape myth acceptance and demographic factors on verdict outcomes. Specifically, this study will examine the impact of jurors' attitudes toward sexual violence, legal attitudes, age, gender, educational attainment, and ethnicity upon individual juror decision-making. The primary focus of this study is to investigate these factors in the context of an intimate partner rape case. Unlike existing literature, which often concentrates on stereotypical rape scenarios, i.e. “date rape,” this study will delve into the specific dynamics and biases that may influence juror decision-making within cases involving intimate partners accused of rape.

Due to the artificial nature of mock trial research, researchers argue that jury decision-making lacks ecological validity, which limits the generalizability of its results (DeMatteo and Anumba, 2009). The tendency to use written vignettes when presenting case information, especially in rape cases, further reduces the ecological validity as the methodology is far removed from the context and environment of an actual trial (Dinos et al., 2015). Thus, the present study sought to build upon existing literature by exploring juror bias using an ecologically improved mock trial paradigm by utilizing more realistic videotaped criminal trial reconstructions of a genuine sexual assault case, according with Willmott et al. (2021) recommendations surrounding how to generate more realistic mock trial research. Traditional jury research is often criticized for its use of university student samples. It is said to lack the depth of life experience real courtroom jurors bring to trial and is, therefore, not an accurate representation of the general population (DeMatteo and Anumba, 2009). To address this criticism, a more representative cross-section of the general population was targeted via a community sample.

It is hypothesized that individuals heightened scores in rape myth acceptance will be more likely to return a not-guilty verdict. Additionally, legal attitudes are hypothesized to significantly affect verdict outcomes, particularly conviction proneness and social justice. Due to mixed previous research findings, no hypothesis was advanced regarding the role of juror demographic variables.

## Method

### Sample

Due to the unique multivariate nature of our anticipated regression model, it was difficult to attain findings from highly similar investigations to base apriori power calculations on. In lieu of this, the authors used the probability estimates from McKimmie et al.'s (2014) investigation of juror gender effects on verdict decisions in rape cases to identify an approximate sample requirement for binary logistic regression model testing. Using a translated Odds Ratio of 2.47 (medium effect) and an estimated 0.5 gender distribution, the power calculation estimated a minimum requirement of 324 cases (*z* =1.96, actual power 0.95). A combination of convenience and snowball sampling methods were employed to recruit UK jury-eligible participants. Inclusion criteria required all participants to be aged 18 to 75, be British citizens, have no serious history of criminality or severe mental health difficulties and be proficient in written and spoken English. The experiment was advertised on online social media platforms (e.g., Facebook, Twitter, LinkedIn), with a weblink directing participants to the Qualtrics-hosted experiment. Initial social media adverts encouraged prospective participants to share the study link among their social networks, as did the study debrief for mock jurors who took part, making clear that no posts or comments were permitted regarding opinions or decisions made regarding the trial. After removing ineligible and incomplete responses, the final dataset consisted of 435 participants (female = 323, male = 112), aged 18–75 (*M*_*age*_ = 33.43, *SD*_*age*_ = 13.05). The most common ethnicity reported was Caucasian (*n* = 374), followed by South Asian (*n* = *33)*, mixed ethnicity *(n* = *10)*, and Black and/or African heritage *(n* = *7*). Other ethnicities reported by participants included Middle Eastern and Arabic heritage, and Native American and Latino heritage (*n* = 11). In relation to educational attainment, 260 participants (60%) held a university bachelor's qualification, or higher, and the remaining 175 respondents (40%) reported their highest academic qualification as being less than a bachelor's degree (i.e., college, vocational or secondary school qualifications).

### Mock trial materials

A transcript of a genuine rape trial, previously heard before a criminal court, was condensed to create a short mock trial reconstruction video that would be shown to mock jurors after they completed the initial pre-trial questionnaires. This case and trial transcript, from which the subsequent trial video was developed, was reviewed by an expert panel of UK criminal justice practitioners (including active police detectives and criminal lawyers) to ensure no key information was missing or misrepresented during the process of reducing the case materials to a testable mock-trial length. The video reconstruction was devised to include case components deemed important to a genuine criminal trial in E&W. These included: the undisputed facts, the complainants account, the defendants account, a shortened version of both the prosecution lawyer and defense lawyers questioning (examination and cross-examination) of the complainant and defendant, key forensic evidence and a summary of the judge's case overview and legal instructions in the case. The final mock trial reconstruction was performed by actors and videotaped. The final video shown to participants was 22 minutes long. A brief written summary of the facts of the case are provided below;

*On the evening of the 8*^*th*^ *February 2014, the complainant, SARAH ADAMS, and the defendant, KYLE WILLIAMS, had arranged to meet at the apartment they once shared so that he could collect some of his possessions. The complainant had lived in the apartment on her own for the past two months since the couple's eight-month relationship had broken down, and the defendant moved out. It was also the first time the couple had seen each other since the end of their relationship, although both had considered they were still friendly and civil to one another at this point. As he had a considerable amount of possessions to collect, the defendant had brought several boxes with him, and the pair had agreed that they would pack the things together over a period of two hours, 6 pm – 8 pm. After an hour, all of the defendant's possessions were packed, so he and the complainant began chatting over coffee and then wine. Approximately two hours later, as the defendant made to leave, the two kissed - something which they both agreed they had consented to. However, this kiss then led to sexual intercourse, and it was at this point that their versions of events differed*. Access to the full written trial transcript can be requested via the author DW.

### Measures

#### Acceptance of modern myths about sexual aggression

The AMMSA scale (Gerber et al., 2007) is a self-report unidimensional 30-item measurement tool developed to subtly measure modern rape myth acceptance and attitudes held toward sexual aggression in diverse populations (e.g., “when it comes to sexual contacts, women expect men to take the lead” and “women often accuse their husbands of marital rape just to retaliate for a failed relationship”). Responses are measured on a seven-point Likert scale (1 = “completely disagree” to 7 = “completely agree”) which are summed up to create a total score (ranging from 30 to 210), with higher scores indicating greater acceptance or endorsement of modern rape myths. The scale has received cross-cultural validation, having been found to demonstrate a reliable factor structure and satisfactory levels of retest reliability (between 0.67 and 0.88). The scale also demonstrated good internal consistency in the present dataset (α = 0.92).

#### Pre-trial juror attitudes questionnaire

The PJAQ (Lecci and Myers, 2008) is a 29-item scale that measures six different sub-types of legal biases that jurors may possess: conviction proneness (5-items, e.g., “criminals should be caught and convicted by ‘any means possible”'), system confidence (6-items, e.g., “if a suspect runs from the police, then he probably committed the crime”), cynicism toward the defense (7-items, e.g., “in most cases where the accused presents a strong defense, it is only because of a good lawyer”), social justice (4-items, e.g., “rich individuals are almost never convicted of their crimes”), racial bias (4-items, e.g., “minorities use the ‘race issue only when they are guilty”), and innate criminality (4-items, e.g., ‘once a criminal always a criminal'). Respondents indicate their agreement with the statements using a five-point Likert scale (1 = “strongly disagree” to 5 = “strongly agree”), with higher total scores reflecting greater prosecution bias. No Cronbach's alpha was calculated for the original scale; however, future work reported an internal consistency of 0.85 (Lundrigan et al., 2016). In the present study, the defendant's race and criminal history were not disclosed. Therefore, the racial bias and innate criminality subscales were not used in the analyses and are not discussed further. The remaining four subscales demonstrated good internal reliability in the present sample (0.80 or higher).

#### Demographic information and verdict decisions

Demographic information was collected regarding mock jurors' self-reported age, gender, ethnicity, and highest level of educational attainment (university bachelor's qualification or above, less than a university bachelor's degree). Due to low cell counts for some of the reported ethnicities by participants, ethnicity responses were recoded into a dichotomy of ‘Caucasian and Black, Asian, and Minority Ethnic' (BAME) for relevant analyses, to allow for some form of comparison. Please note the term BAME is historically used to denote non-Caucasian British residents and citizens whose ethnic group is described as being of Black Caribbean or African descent or South East Asian descent (typically of Indian or Pakistani heritage). Finally, participants indicated their verdict decisions (in response to “How do you find the defendant, Kyle Williams, on the allegation that he raped the complainant, Sarah Adams?”) through a closed-question response (guilty or not guilty).

### Procedure and analysis

The present study adopted an online mock trial paradigm whereby a short video of a mock trial reconstruction depicting an intimate partner rape case was presented to individual mock jurors. Case information and trial reconstruction were developed from the trial transcript of a genuine real rape allegation that was previously examined in court. A cross-sectional design was employed, in which all participants completed the same battery of surveys at a single time point. To mitigate risks of common method variance associated with cross-sectional approaches (see Spector, 2019), the authors employed numerous procedural remedies, as implemented by Mojtahedi et al. (2021). Firstly, instructions emphasized that each set of questions measured a different construct to psychologically distance the scales from one another, an approach shown to reduce CMV (Podsakoff et al., 2003). Additionally, the authors attempted to minimize social desirability effects and evaluation apprehension by informing participants that there were no correct answers. The experiment was hosted on an online survey platform (*Qualtrics*). After providing informed consent, participants were instructed to complete a series of questionnaires (demographic questions, AMMSA, and PJAQ). Next, participants were then informed they would be watching a mock trial reconstruction video of an alleged rape that occurred between two people who were in an existing intimate relationship, based on evidence presented from the real trial. After the video had elapsed, participants were asked to provide a verdict decision based on the evidence they had observed. All statistical analyses were performed using SPSS^®^ 24.0 (IBM Corporation, Armonk, NY, USA) for Windows^®^/Apple Mac^®^. For all logistic regression models, preliminary analyses indicated that there were no multivariate outliers, and multicollinearity was unlikely to be a problem (in accordance with O'Brien, 2007; see VIF/Tolerance values in Table 2).

## Results

Descriptive statistics and correlations for all continuous variables are presented in Table 1.

**Table 1:** Descriptive statistics and correlation coefficients for continuous variables (N = 435).

Variables	Age	AMMSA	CON	CP	CYD	SJ
Age	–					
AMMSA	−0.087	–				
CON	0.008	0.329^**^	–			
CP	−0.107^*^	0.174^**^	0.522^**^	–		
CYN	−0.159^**^	0.261^**^	0.357^**^	0.437^**^	–	
SJ	−0.109^*^	−0.008	0.129^**^	0.19^**^	0.38^**^	–
Mean	33.43	80.94	16.04	14.10	21.78	13.39
Std Dev	13.05	25.82	3.65	3.47	4.30	2.39
Range	18–75	30–176	6–27	5–25	7–33	7–19

AMMSA, Acceptance of Modern Myths about Sexual Aggression; CON, Confidence in the criminal justice system; CP, Conviction proneness; CYN, Cynicism toward the defense; SJ, Social justice.

^*^ = < 0.05, ^**^ = < 0.001.

Overall, 71.0% (*N* = 309) of participants returned a guilty verdict. Frequency distributions suggest that guilty verdicts were more common among female participants (74.9%, *N* = 242) compared to male participants (59.8%, *N* = 67), Caucasian participants (74.1%, *N* = 277) compared to BAME participants (52.5%, *N* = 32), and university educated participants (73.7%, *N* = 129) compared to non-university educated participants (69.2%, *N* = 180).

Binary logistic regression analysis was conducted to assess the predictive effects of our chosen juror variables (age, gender, educational attainment, confidence in the criminal justice system, conviction proneness, cynicism toward the defense, social justice, and AMMSA) upon verdict decisions (Guilty/Not-Guilty). A hierarchical approach was used to determine whether any significant associations between verdict decision and the demographic grouping variables existed after accounting for differences in pre-trial attitudes (pre-trial juror legal attitudes and rape myth beliefs).

In the first step, the demographic variables (gender, ethnicity, education and age) were entered. The model was statistically significant, [χ^2^ (*df* = 4, *N* = 433) = 20.21, *p* < 0.001, Cox and Snell R2 = 0.046, Nagelkerke R2 = 0.065], correctly classifying 71.8% of cases into the correct outcome category. Only Ethnicity (OR = 2.7, *p* < 0.001) and Gender (OR = 0.52, *p* = 0.006) made significant contributions to the model (see Table 2).

**Table 2:** Binary logistic regression models of factors influencing verdict outcomes (*n* = 435).

Variables	*B*	*SE*	OR (95% CI)	Tolerance/ VIF
Step 1				
Age	−0.01	0.01	0.99(0.98/1.01)	0.94/1.07
Male	−66	0.24	0.52 (0.33/0.83)^**^	1/1
University degree	0.20	0.23	1.22 (0.79/1.91)	0.98/1.02
Caucasian	0.99	0.30	2.7(1.51/4.82)^***^	0.95/1.05
Step 2				
Age	−0.01	0.01	0.99 (0.97/1.01)	0.91/1.01
Male	0.21	0.27	0.81 (0.48/1.34)	0.92/1.09
University degree	−0.49	0.25	1.64 (1.01/2.67)^*^	0.96/1.05
Caucasian	0.69	0.34	2 (1.03/3.87)^*^	0.88/1.14
AMMSA	−0.04	0.01	0.96 (0.95/0.98)^***^	0.76/1.31
CON	0.04	0.04	1.04 (0.97/1.13)	0.64/1.57
CP	0.07	0.04	1.07 (0.99/1.17)	0.64/1.57
CYD	−0.07	0.04	0.94 (0.88/1.00)	0.65/1.53
SJ	0.10	0.06	1.11 (0.10/1.24)	0.8/1.25

AMMSA, Acceptance of Modern Myths about Sexual Aggression; CON, Confidence in the criminal justice system; CP, Conviction proneness; CYN, Cynicism toward the defense; SJ, Social justice *SE*, Standard Error; OR, Odds Ratio; 95% CI, Confidence Interval.

^*^p < 0.05.

^**^p < 0.01.

^***^p < 0.001.

The pre-trial attitude predictors (AMMSA, confidence in the criminal justice system, conviction proneness, cynicism toward defense, and social justice) were entered in the second stage [χ^2^ (*df* = 5, *N* = 433) = 62.79, *p* < 0.001]. The overall model remained significant [χ^2^ (*df* = 9, *N* = 433) = 83, *p* < 0.001, Cox and Snell R^2^=0.174, Nagelkerke R^2^ = 0.249], correctly classifying 75.5% of cases. In the final model, only three variables (ethnicity, AMMSA, and education) were able to significantly predict verdict decisions. That is, participants who were Caucasian (OR = 2, *p* = 0.04), scored low in rape myth endorsement (OR = 0.96, *p* < 0.001) and those who were university educated (OR = 1.64, *p* = 0.048), were more likely to return *guilty* verdicts. The association between rape myth acceptance and verdict decisions is exemplified further through the large observable difference (*d* =0.89) in mean AMMSA scores between participants returning guilty (*M*_AMMSA_ = 74.75, Std Dev_AMMSA_ = 23.19) and not-guilty verdicts (*M*_AMMSA_ = 96.1, Std Dev_AMMSA_ = 25.78).

**Figure 1:**
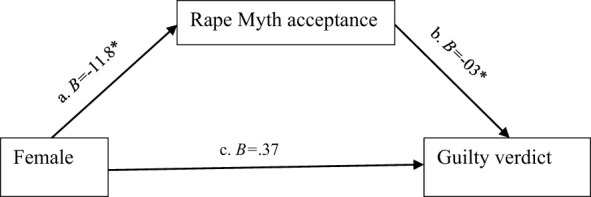
Mediation model for verdict decision. *p < 0.001.

After controlling for pre-trial attitudes, gender was no longer a significant predictor of verdict decision, suggesting that male participants' observed reluctance to convict the defendant was primarily a result of differences in pre-trial attitudes, namely rape myth beliefs—confirmed given that it was the only significant *attitudinal* predictor of guilty verdict selections. A mediation analysis was carried out using Hayes's (2012) Macro Process via the bootstrapping method (5,000 re-samples), to determine whether rape myth acceptance had a mediational effect on the relationship between gender and guilty verdicts (see Figure 1). Indirect effects (mediation) were only considered significant if the respective corrected 95% CI excluded zero.

The results showed that the total effect between gender and verdict decision was not statistically significant (B = 0.37, *p* = 0.147); however, female participants reported significantly lower levels of rape myth acceptance (B = −11.8, *p* < 0.001) and rape myth acceptance was significantly associated with not-guilty verdict decisions (B = −0.03, *p* < 0.001). When rape myth acceptance entered the relationship between gender and verdict decision, the indirect effect of gender on verdict decisions through rape myth acceptance was significant, the bias corrected 95% CI was 0.4, and bootstrapped CI 95% = 0.2 to 0.66, which excluded zero.

## Discussion

The present study sought to investigate the relationship between legal attitudes, modern rape myth acceptance and demographics upon verdicts, specifically within the context of an intimate partner rape trial. Results identified that individuals who endorsed rape myths to a greater extent were more likely to yield not-guilty verdicts compared to individuals low in rape myth acceptance. These results appear to provide support for the Story Model of juror decision-making (Pennington and Hastie, 1992). In the absence of definitive evidence, a hallmark of IPR, mock jurors appear to use rape myths as a narrative framework to interpret ambiguous case information. Mock jurors' legal attitudes could not significantly predict verdict outcomes, suggesting that when “filling in the blanks” of a complex encounter, mock jurors prioritize pre-existing social schemas over abstract legal principles to construct a plausible version of events. Among demographic factors, jurors‘ ethnicity, gender, and educational attainment were found to predict their verdicts; however, after controlling for pre-existing attitudes, only ethnicity and education remained significant.

In light of previous academic research, the present study provides a unique, ecologically improved contribution to the discipline that builds on prior investigations. In terms of rape myth acceptance, results support those of prior research that identified a direct relationship between rape myth acceptance and overall verdicts. More specifically, individuals high in rape myth acceptance are more likely to return a not-guilty verdict due to their inaccurate and distorted beliefs regarding rape and victims of rape (McGee et al., 2011; Hammond et al., 2011; Willmott and Woodhams, 2026). Thus, implying that defendants accused of non-stereotypical rapes are less likely to be convicted (Ellison and Munro, 2009). This was challenged in the present study as mock jurors were presented with an intimate partner rape scenario. The use of a non-stereotypical rape situation allowed the research to probe an accurate representation of an individual's rape myth acceptance. The broader literature has highlighted the ambiguity of consent, particularly between current or former intimate partners, as a significant influence on juror decision-making, as was apparent in the present scenario. Yet, results show that 72.8% of mock jurors returned a guilty verdict, contradicting existing research in so far as mock jurors appeared more willing in this case, to convict the accused than is typically found in mock jury research utilizing acquaintance rape scenarios. Although the wider jury literature suggests IPR cases are less likely to result in conviction, the high conviction rate in the current study may still be explained by the Story Model of juror decision-making. The theory suggests that while IPR is inherently ambiguous, the mock jurors who returned guilty verdicts were able to construct a narrative with high coherence and coverage that overrode the stereotypical scripts associated with intimate partner abuse. This suggests that while rape myths are a powerful tool for filling gaps, if the evidence of the victim's lack of consent was presented clearly, jurors could build a narrative of guilt that felt more complete than a narrative of consensual sex.

The analysis also revealed that gender was initially identified as a predictive factor of verdict outcomes. However, this relationship was no longer significant once pre-existing attitudes, such as rape myth acceptance, were incorporated into the model. This suggests an indirect relationship between gender and verdict decisions, whereby male jurors with higher levels of rape myth endorsement are more likely to deliver not-guilty verdicts. These findings imply that addressing rape myth acceptance may mitigate gender disparities in the decision-making process. One potential strategy to address this issue is to incorporate rape myth education into the jury selection process. Whilst this approach is frequently proposed by academics (Hudspith et al., 2024; Temkin and Krahé, 2008; Willmott and Hudspith, 2024), the current paper proposes a more targeted intervention directed toward those identified as having high levels of rape myth endorsement, exclusively.

Past research posits that pre-existing legal attitudes may predict verdict outcomes (Lundrigan et al., 2016; Willmott et al., 2026a). More specifically, regarding the PJAQ scale used in the present study, research suggests that greater cynicism toward the defense, conviction proneness, and system confidence would be predictive of guilty verdicts (Farrell et al., 2013; Lecci and Myers, 2009; Sandys et al., 2018). However, the present study conflicts with past research, failing to show any association between pre-trial legal attitudes and verdict decisions. One explanation is the complexity and ambiguity of intimate partner rape cases. Issues regarding an apparent lack of consent and conflicting accounts may lead jurors to justify a defendant's actions as acceptable, even when they contradict their beliefs. Our conflicting findings could also be attributed to the confounding relationship rape myth acceptance has with the aforementioned legal attitudes. As shown in Table 1, rape myth acceptance was positively associated with conviction-oriented attitudes (greater confidence in the system, conviction proneness, and cynicism toward the defense). Thus, it is possible that for jurors holding such prosecution biases, negative attitudes toward rape victims may override their inclinations to convict suspected offenders.

Previous research considering ethnicity primarily focused on the defendant/victim's race rather than a juror's. Consequently, the current report provides a preliminary insight into the associations between juror ethnicity and juror decision-making in criminal trials. Present research findings indicate that Caucasian mock jurors are significantly more likely to return a guilty verdict compared to the BAME subsample. Given that the race of the defendant and victim was not salient, despite names reflecting Caucasian/western cultures, these findings arguably depart from existing literature that suggests minority defendants are more likely to be found guilty and awarded harsher sentences (Mitchell et al., 2005; Bottoms et al., 2004). Furthermore, no evidence of same-race leniency was observed, further challenging existing research on ethnicity in the courtroom. While the current research findings highlight significant demographic associations, they should be interpreted with caution. The observed differences between Caucasian and BAME jurors may reflect broader variations in societal experiences or attitudes toward intimate relationships. However, as this study did not directly measure cultural norms or specific rape-related beliefs, these connections remain speculative. Although some cross-cultural research suggests varying levels of acceptance regarding physical aggression between partners in different global contexts (Archer, 2006), it cannot be assumed that the current BAME subsample endorses “non-Western” cultural beliefs. Therefore, these findings should be viewed as preliminary demographic trends rather than definitive evidence of cultural influence, highlighting the need for future research to utilize a larger ethnically diverse sample and direct measurement of cultural attitudes.

The present study identified educational attainment as a significant predictor of juror decision-making, despite considerable inconsistencies in the existing literature (McCabe et al., 2010; Hosch et al., 2011; Willmott, 2018). Mock jurors with a university degree were more likely to convict than non-university-educated equivalents. There is no empirically acknowledged relationship between educational attainment and verdict decisions, so explanations muay originate from student and community sample comparisons. Previous research argues that students make more calculated, informed decisions (Hosch et al., 2011). Thus, when concerned with an ambiguous intimate partner rape allegation, more educated mock jurors evaluate evidence meticulously to comprehend case information. Subsequently, they may have been more likely to base their verdicts on evidence rather than on general beliefs.

### Study strengths and limitations

Traditional jury research has consistently adopted mock trial designs far removed from the context of a genuine courtroom. For example, often utilizing written vignette “mock trial scenarios” that lack ecological validity due to their artificial nature (Dinos et al., 2015). To address this issue, the present study used more realistic videotaped criminal trial reconstructions of a genuine rape trial. Thus, increasing the ecological validity of the present research findings. This methodology also reduces the risk of idiosyncratic interpretations of case information, often present in written vignette experiments, as information is presented to jurors in a standardized format (Lieberman et al., 2016). The ecologically improved methodology is a significant strength of the current research as it increases the reliability and generalizability of the results produced and goes someway toward adopting the six recommendations advanced by Willmott et al. (2021) advocating for more policy-relevant mock jury rape trial research. Yet, to maintain experimental control and adhere to the research timeline, trial materials were significantly condensed, necessitating components like live cross-examination and comprehensive judicial instructions. Consequently, the mock trial paradigm did not fully reflect the complexities of real-world criminal proceedings – a study limitation important to recognize. Furthermore, consistent with much prior jury research, the current study design did not include group deliberation and assessed individual jurors' verdict selections made in isolation. Despite utilizing an ecologically improved mock-trial paradigm, the present study remains an imperfect representation of a real-world courtroom and its procedures. This methodological limitation undoubtedly limits the generalizability of the findings and thus future research should seek to both include jury deliberation to improve realism to genuine trial procedures. It can be argued that certain predictor variables may differ in their influence before and after deliberation, as recently reported by Willmott et al. (2026b) in their live quittance rape trial enactment study. Indeed, recent qualitative studies that included and analyzed jury deliberations found rape myths remain a core part of deliberative discussions underpinning group verdict outcomes (Richardson et al., 2025; Weare et al., 2025). Therefore, future research seeking to examine the important of juror traits in IPR should include group deliberations and measure verdict outcomes before and after deliberation. Nonetheless, the use of a video-taped trial format adopting in this study represents a significant methodological step forward from most existing research relying solely on written transcripts and vignettes.

An additional strength of the present research is the diverse community sample. As previously mentioned, existing jury research typically uses student samples, which are argued to lack the relevant life experience of a legitimate juror (DeMatteo and Anumba, 2009). Therefore, the present study contributes to prior research by investigating juror biases using a representative sample. To be specific, the present sample consists of individuals varying in age and in educational attainment. Once again, this furthers the generalizability of the study findings as the sample more accurately reflects the general population from which potential jurors are selected (Coolican, 2019). However, it is important to acknowledge that the recruitment strategy adopted in this study may have introduced selection bias into the sample. Recruitment through convenience and snowball sampling may have attracted individuals with strong pre-existing attitudes toward sexual violence, higher levels of education or specific criminal justice beliefs. Furthermore, the sample did not fully represent the general population, as it was heavily female-skewed and predominantly Caucasian. Whilst the inclusion of community members was a methodological advantage, the fact that only 14% of the current sample identified as BAME underscores the need for more ethnically diverse samples in future research to better capture the breadth of perspectives found in a diverse jury pool.

### Implications and future directions

The present study provides a unique contribution to understanding the effects of juror traits and biases on final verdict outcomes. Due to the confirmation that rape myth acceptance directly impacts verdict decisions within an ecologically improved mock trial paradigm, results of previously criticized research are here corroborated by the present study results. As rape myth acceptance has been highlighted as a significant predictor of verdict outcome, the present study informs practitioners and policymakers in terms of reform. For instance, there is a need to review whether real rape trial juries can indeed be considered fair and impartial and, therefore, securitize the use of juries in future rape trials. Jury reform could address some of the issues found in this study and may include training individual jurors pre-trial and/or in-trial in an effort to reduce rape myth bias and other case-specific biases (for a detailed review see Willmott and Hudspith, 2024). Indeed, some recent efforts to evaluate the efficacy of rape myth debunking interventions yield some promising results (see Hudspith et al., 2024). Future research should also seek to investigate the role of additional psychological variables in jurors decision making, that the current study did not account for. Examples include, “Dark Tetrad” (e.g. psychopathy, Machiavellianism), victim empathy and moral disengagement. Existing evidence suggests these traits significantly influence attitudes toward vindictive rape (Navas et al., 2022; Veggi et al., 2026), with rape myths found to be highly correlated with these traits (Ioannides and Willmott, 2023; Willmott and Widanaralalage, 2024). Additionally, exploring motivations related to altruistic or costly punishment could provide deeper insight into how jurors assess defendant culpability (Hanson, 2020). By examining these psychological factors, future studies can provide a more comprehensive understanding of the factors that influence juror decision-making.

## Conclusion

In conclusion, the present study identified rape myth acceptance, ethnicity and educational attainment as significant predictors of verdict outcomes in IPR. These factors distinguished between individuals likely to return a guilty verdict and those likely to return a not guilty verdict, indicating that specific pre-trial juror characteristics *do* appear to influence the verdicts that jurors ultimately reach. Thus, future research is encouraged to incorporate real-world practice to investigate the aforementioned variables within further enhanced mock trial methodologies and more realistic settings to directly examine the reliability of the present results in real-world jury decision-making. It is suggested that future researchers record preferred verdicts before and after group deliberations to help better understand how juror biases affect decision-making and final verdicts, similar to recent efforts by Willmott et al. (2026b).

## Data Availability

The raw data supporting the conclusions of this article will be made available by the authors, without undue reservation.
